# T cell receptor excision circles are potential predictors of survival in adult allogeneic hematopoietic stem cell transplantation recipients with acute myeloid leukemia

**DOI:** 10.3389/fimmu.2022.954716

**Published:** 2022-09-20

**Authors:** Anna Söderström, Sofie Vonlanthen, Kerstin Jönsson-Videsäter, Stephan Mielke, Hannes Lindahl, Johan Törlén, Michael Uhlin

**Affiliations:** ^1^ Department of Clinical Science, Intervention, and Technology, Karolinska Institutet, Stockholm, Sweden; ^2^ Department of Clinical Immunology and Transfusion Medicine, Karolinska University Hospital, Stockholm, Sweden; ^3^ Cell Therapy and Allogeneic Stem Cell Transplantation, Karolinska Comprehensive Cancer Center, Karolinska University Hospital, Stockholm, Sweden; ^4^ Department of Clinical Neuroscience, Karolinska Institutet, Stockholm, Sweden

**Keywords:** TREC, immune reconstitution, allogeneic stem cell transplantation, KREC, lymphocyte neogenesis, survival, AML

## Abstract

**Background:**

Lymphocyte neogenesis from primary lymphoid organs is essential for a successful reconstitution of immunity after allogeneic hematopoietic stem cell transplantation (HSCT). This single-center retrospective study aimed to evaluate T cell receptor excision circles (TREC) and kappa-deleting recombination excision circles (KREC) as surrogate markers for T and B cell recovery, as predictors for transplantation-related outcomes in adult acute myeloid leukemia (AML) patients.

**Methods:**

Ninety adult patients diagnosed with AML and treated with HSCT between 2010 and 2015 were included in the study. TREC and KREC levels were measured by quantitative PCR at 1, 3, 6, and 12 months after transplantation.

**Results:**

Overall, excision circle levels increased between 3 and 6 months post-HSCT for TREC (p = 0.005) and 1 and 3 months for KREC (p = 0.0007). In a landmark survival analysis at 12 months post-HSCT, TREC levels were associated with superior overall survival (HR: 0.52, 95% CI: 0.34 - 0.81, p = 0.004). The incidence of viral infections within the first 100 days after transplantation was associated with lower TREC levels at 6 months (p = 0.0002). CMV reactivation was likewise associated with lower TREC levels at 6 months (p = 0.02) post-HSCT. KREC levels were not associated with clinical outcomes in statistical analyzes.

**Conclusions:**

Results from the present study indicate that TREC measurement could be considered as part of the post-HSCT monitoring to identify AML patients with inferior survival after transplantation. Further prospective studies are warranted to validate these findings.

## Introduction

Allogeneic hematopoietic stem cell transplantation (HSCT) is a common treatment alternative for several hematological malignancies, metabolic diseases, and inborn errors of immunity. Patients with acute myeloid leukemia (AML) comprise over a third of all HSCT patients in Europe in 2018 (1). The introduction of reduced conditioning therapy alternatives and improved supportive care has gradually made the treatment available for a growing group of older patients (2–4). Though the treatment can be curative it is also associated with severe risks such as transient immunodeficiency with susceptibility to infections, graft versus host disease (GvHD), relapse, and rejection (5). For the treatment to be successful, reconstitution of the immune system is of utmost importance. Delayed recovery of the transplanted immune system can increase the risk of transplant-related complications, and patients are thoroughly monitored after transplantation (6–8).

Immune reconstitution of different subtypes of immune cells occurs successively and at different time points after HSCT. While cells of the innate immune system recover within weeks from the transplantation, cells of the adaptive immune system can take months to years to fully recover regarding quantity, function, and competence (9). Mature lymphocytes in the donor graft will proliferate in the recipient early after transplantation but have a limited repertoire and competence (10, 11). More important for long-term pathogen defense, tumor control, and self-tolerance is the neogenesis of naïve lymphocytes from primary lymphoid organs (12).

To assess the neogenesis of lymphocytes, naïve lymphocyte subsets can be quantified by flow cytometry (13–15). Another proposed method to measure lymphocyte neogenesis is the quantification of T cell receptor excision circles (TRECs) and B cell kappa-deleting recombination excision circles (KRECs). These excision circles are circular DNA fragments that are by-products generated during the V(D)J recombination in the process of T and B cell receptor formation (16). Studies have shown excision circles do not replicate and are diluted with each cell division and a strong positive correlation between TRECs and recent thymic emigrants (15, 17, 18). TREC and KREC measurement has been applied in various settings as proxy markers to evaluate the *de novo* production of lymphocytes, e.g., screening for primary immunodeficiencies and monitoring of secondary immunodeficiencies such as HIV (19). More recently, studies have investigated applying this analysis to the monitoring of adaptive immune reconstitution after HSCT (10, 17, 20–25). Most previous studies on this topic are performed on quite heterogeneous study populations, comprising both children and adults with different treatment indications for HSCT.

In the present study, we evaluated the post-transplant monitoring of TREC and KREC in a cohort of adult AML patients, the most common indication for HSCT. We aimed to explore how different transplant-related variables are associated with adaptive immune reconstitution, and if TREC and KREC measurements could be used to predict risk for transplant-related events.

## Materials and methods

### Study population

Ninety adult AML patients who underwent HSCT between 2010 and 2015 at Karolinska University Hospital in Stockholm, Sweden, were included in the study. The study was approved by the regional review board of ethics in Stockholm (DNO: 2021-00977). Patients were classified into risk groups according to cytogenetic abnormality (26).

### HSCT features and definitions

Patients and donors were typed on HLA class I and II by molecular high-resolution typing (27).

The conditioning regimen was chosen according to standard protocols at the transplant center, depending on patient age, comorbidities, and donor type. Briefly, younger and otherwise healthier subjects were administered myeloablative conditioning (MAC), which consisted of fludarabine in combination with busulfan in myeloablative doses. Reduced-intensity conditioning (RIC) included fludarabine in combination with lower doses of busulfan or treosulfan (28). Patients with an unrelated donor (URD), or male patients with a female sibling donor (SIB), were given anti-thymocyte globulin (ATG) intended for *in vivo* T cell depletion in addition to the conditioning regimen to decrease the risk of rejection and GvHD (29, 30).

GvHD prophylaxis consisted of cyclosporine + methotrexate or tacrolimus + sirolimus. The two prophylaxis options were not significantly associated with lymphocyte reconstitution according to a previous randomized study conducted at our center, and hence we did not focus on this matter in the present study (25). Acute and chronic GvHD was assessed by treating physicians at regular follow-up or admissions to the hospital. Acute GvHD was graded from grade 0 (not present) to IV, according to previous mentioned criteria (31). Acute GvHD Grade II to IV was considered severe aGvHD. Chronic GvHD was graded according to the National Institute of Health criteria for clinical trials (32).

### TREC and KREC measurement

Peripheral blood samples were collected at 1, 3, 6, and 12 months after HSCT, with a time window of +/- 10 days at earlier timepoint and 30 days at later time points. An automated magnetic bead-based extractor (Arrow) was used for CD3^+^ and CD19^+^ cell isolation, according to the manufacturer’s protocol (NorDiag, Bergen, Norway). DNA was extracted from the separated cells by a standard protocol and stored at -20°C until analysis. δRec-ψJα signal-joint TREC or KREC, and the control gene β-actin were measured using duplex quantitative PCR (ImmunoI IVD, Stockholm, Sweden) with minor adjustments to what was previously described (33). The amount of TREC in CD3^+^ cells and KREC in CD19^+^ cells were presented as a ratio between excision circles and β-actin, calculated with the ΔCt method (34).

### Statistical analysis

Patient characteristics are presented as relative or absolute frequencies, continuous variables are presented as median with range. Continuous variables were compared using the Mann-Whitney U test. Spearman correlation was used. Landmark survival analyzes were performed at 6 and 12 months post-HSCT. Overall survival (OS) and relapse-free survival (RFS) were depicted in Kaplan-Meier graphs and comparisons between subgroups of patients were calculated with the log-rank test. In uni- and multivariable models, Cox proportional hazards ratio was used to evaluate the effect of explanatory variables on time-dependent outcomes. Multiple logistic regression was used to investigate the association of the variables with time-independent outcomes. Variables included in the multivariable analysis were variables that were significant in the univariable analysis. A two-tailed p-value < 0.05 was considered significant. Since this is an exploratory study, p-values were not corrected for multiple testing.

Samples, where no TREC or KREC could be detected, were assigned a value below the lowest measured value in the cohort, for statistical purposes. Due to the right-skewed distribution of TREC and KREC data, log transformation was applied, which was only partially effective in approximating normality. Data analysis was performed using Graphpad Prism version 9 (Graphpad Software, San Diego, CA, USA) and R version 4.1.2.

## Results

### Patient characteristics

**Figure 1:**
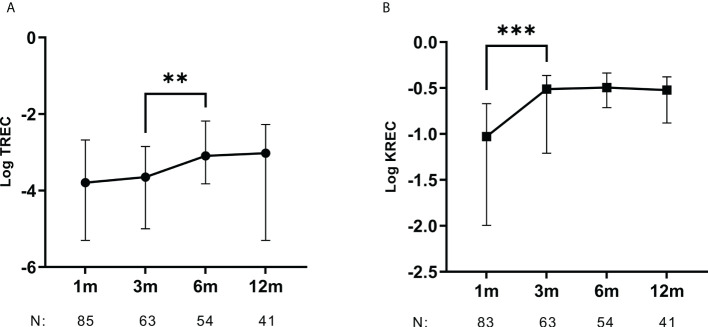
TREC and KREC levels increase within the first months after HSCT. Kinetics for **(A)** TREC and **(B)** KREC levels from 1 to 12 months after HSCT. Median with whiskers for interquartile range. N: Number of samples analyzed at each time point. Mann-Whitney U-test. **p < 0.01, ***p < 0.001.

Patient characteristics are described in **Table 1**. Patients that had at least one sample available for analysis of TREC and KREC were included in the study (n = 90), the number of analyzed samples at each time point is shown in **Figure 1**. Clinical records were evaluated, and no systematic selection bias on patients that had samples available for analysis was found. The median follow-up time in the cohort was 80 months (range: 0-140 months). Of note, only 5 out of 90 patients received a bone marrow graft, and the remainder received peripheral blood stem cells. As a result of the small numbers, no statistical analysis could include this variable.

**Table 1:** Patient characteristics.

Characteristics	N=90
Age, *median* (range)	52 (18–71)
CD34+ cell dose x10^6^/kg, *median* (range)	7.3 (1.4–14.2)
Cytogenetic risk classification, *n (%)*	
Low risk	8 (9)
Intermediate risk	51 (57)
High risk	31 (34)
Sex, *n* (%)	
Female	41 (46)
Male	49 (54)
Graft source, *n* (%)	
PBSC	85 (94)
BM	5 (6)
Donor type	
URD	67 (74)
SIB	23 (26)
Conditioning therapy, *n* (%)	
MAC	35 (39)
RIC	55 (61)
ATG, *n* (%)	
Yes	66 (73)
No	24 (27)
ABO match, *n* (%)	
Yes	42 (47)
No	48 (53)
aGvHD, grade, *n* (%)	
No aGvHD	30 (33)
Grade I	30 (33)
Grade II	26 (29)
Grade III-IV	3 (3)
Unknown	1 (1)
cGvHD, *n* (%)	
Yes	29 (32)
No	52 (58)
Unknown	9 (10)
CMV serostatus pre HSCT, *n* (%)	
Positive	67 (74)
Negative	23 (26)
CMV reactivation (out of CMV positive), *n* (%)	
Yes	50 (75)
No	12 (25)
Sex match (Donor-Patient), *n* (%)	
Female-Female	26 (29)
Male-Female	15 (17)
Female-Male	7 (8)
Male-Male	42 (47)
Donor	
Age, *median* (range)	32.5 (19–66)
Female sex, *n* (%)	33 (37)
Male sex, *n* (%)	57 (63)
Positive CMV serostatus, *n* (%)	44 (49)
Negative CMV serostatus, *n* (%)	46 (51)

### Baseline variables associated with TREC and KREC levels

Since the experience of monitoring excision circles in this setting is limited, we initially evaluated associations found in previous studies on our data. Included explanatory baseline variables for excision circles above the median in the multivariable analysis are shown in **Table 2**. Non-significant variables in the univariate analysis not shown in the table were: infused CD34+ cells/kg, patient sex, cytogenetic risk group and female donor to male recipient as opposed to all other sex matches.

**Table 2:** Patients with ATG in their conditioning regimen had lower TREC values post-HSCT.

	OR	95% CI	p-value
**TREC**	** **	** **	** **
1 month			
ATG	35.6	3.9 - 324	**0.002**
Age donor	1	0.96 - 1.06	0.87
ABO major mm	0.59	0.20 – 1.78	0.35
3 months			
ATG	30	3.5 – 257	**0.002**
Age donor	1.03	0.97 - 1.09	0.35
6 months			
ATG	16.3	1.44 - 185	**0.02**
Age donor	1.02	0.97 - 1.08	0.45
Age patient	0.95	0.90 - 0.99	**0.02**
12 months			
ATG^a^	5.54	1.10 – 30.5	**0.049**
**KREC**			
1 month	NS		
3 months	NS		
Conditioning (MAC)^a^	0.25	0.09 - 0.71	**0.01**
6 months	NS		
12 months	NS		

Bold values indicate significant p-values (< 0.05). NS, Not significant.

Both TREC and KREC levels gradually increased over time after HSCT. For TREC, levels increased significantly between samples taken 3- and 6-months post HSCT (p = 0.005) and for KREC between 1 and 3 months (p = 0.0007) (**Figure 1**).

We found no significant inverse correlation between age and TREC levels at any time point (**Supplementary Figure 1**). In the univariable analysis with logistic regression, we observed a weak negative association between age and TREC levels above the median at 6 months post-HSCT (OR: 0.95, 95% CI: 0.90-0.99, p = 0.02), but not at the remaining time points (**Table 2**). Patient age was not correlated with KREC levels at any time point in our material.

The conditioning regimen was not associated with TREC levels at any time point. Patients that received RIC were more likely to have low KREC levels at 3 months, but not at other time points. Patients who received ATG in addition to their conditioning therapy had significantly lower TREC levels at all measured time points, although most significant at the earlier time points (**Table 2**). The factors ATG and donor type: unrelated donor were correlated in all subjects but one. Henceforward in the study, only ATG was included in statistical analysis to avoid multicollinearity. ATG was not associated with KREC levels at any time point.

### GvHD

The presence of aGvHD (onset before day 100) was not associated with TREC or KREC levels at later time points (6 and 12 months). The TREC or KREC values at earlier time points were not associated with later development of cGvHD (data not shown).

### Infections

**Figure 2:**
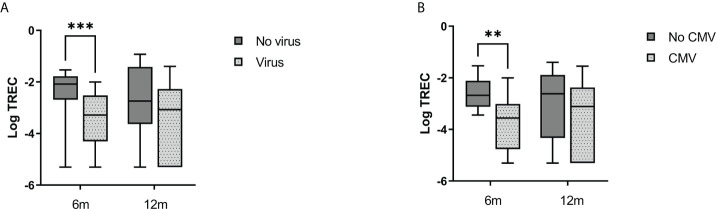
Incidence of viral infections and CMV reactivation was associated with lower TREC levels. **(A)** Virus (light gray boxes) indicated at least one viral infection with a verified pathogen within the first 100 days from HSCT. **(B)** CMV (light gray boxes): CMV-reactivation in CMV seropositive patients within the first 100 days from HSCT. Median with boxes for interquartile range and whiskers for range. Mann-Whitney U-test. **p < 0.01, ***p < 0.001.

Patients who experienced at least one viral infection with a verified pathogen within 100 days from HSCT had significantly lower TREC levels at 6 months post HSCT compared to those who did not (**Figure 2**). When we assessed infections of the herpes virus family only, the association with lower TREC levels persisted. For CMV seropositive recipients who experienced CMV reactivation within 100 days from HSCT, we observed lower TREC levels at 6 months after HSCT (**Figure 2**). CMV reactivation was defined as detectable copies of CMV DNA in patient serum, with or without CMV disease. The median time from HSCT to first viral infection and CMV reactivation was 27 days and 32 days, respectively.

Bacterial infection with a verified pathogen within 100 days from transplantation was not associated with TREC levels at later time points. Incidence of early infections was not associated with KREC levels.

### Survival

**Figure 3:**
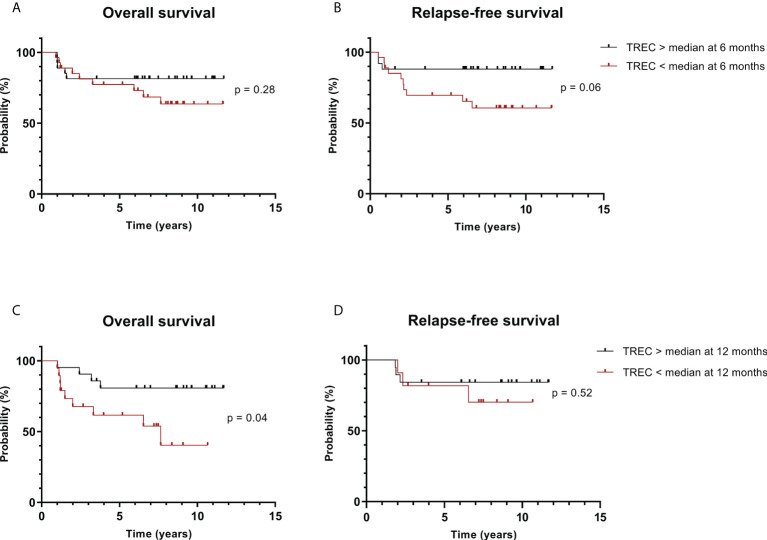
Patients with higher TREC levels have superior overall survival. Patients were divided into groups according to TREC level above or below the median in samples taken at **(A, B)** 6 months post-HSCT; **(C, D)** 12 months post-HSCT. Kaplan-Meier plots with p-value from log-rank test.

To assess if TREC and KREC levels were associated with survival, patients were divided into two groups with excision circles higher or lower than the median at all measured time points after HSCT and a landmark survival analysis was performed. The subgroup with higher TREC levels at 12 months after HSCT had a higher probability of overall survival (OS), no significant difference was found at other time points (**Figure 3**). For relapse-free survival (RFS), the log-rank test did not demonstrate any significant difference between the groups with higher or lower TREC levels (**Figure 3**). We also performed a Cox proportional hazards ratio calculation as a univariable analysis to evaluate various explanatory variables for OS. Here, the TREC level was handled as a continuous variable instead of a dichotomous one. The test demonstrated that higher TREC levels at 12 months post-HSCT were associated with superior OS (HR: 0.52, 95% CI: 0.34 – 0.81, p = 0.004). No other variables reached the significance level in the univariable analysis, and hence no multivariable analysis was performed (**Supplementary Table 1**). For the 25 patients who died before censoring, the cause of death was relapse (n = 14), HSCT-related (infection, GvHD) (n = 7), or unknown (n = 4). When the cohort was divided according to KREC levels, no significant difference was seen in overall survival (data not shown).

## Discussion

Lymphocyte neogenesis from primary lymphoid organs is essential for a successful allogeneic HSCT. The road to full immune reconstitution is a protracted and complex process under which various factors influence on. An impaired immune reconstitution can result in infections, relapse, or other adverse transplant-related outcomes (6–8). As HSCT implies a high risk for complications, a wider set of tools to monitor patients after HSCT is warranted. It would aid the identification of patients at risk for adverse outcomes, and even point out where measures for prevention or preemptive interventions may be considered. This study investigated the use of TREC and KREC monitoring after HSCT in adult AML patients only. Most previous studies have included both adult and pediatric patients with both malignant and benign indications for HSCT. Patients with AML comprise over a third of all HSCT recipients, and the group is growing with older patients eligible for transplantation. By studying this homogenous group of patients, we aimed at excluding confounders such as the obvious influence of age in pediatric patients with a higher thymus function, pathology of disease, and influence of various induction therapies.

We aimed to explore how different transplant-related variables are associated with adaptive immune reconstitution, and if TREC and KREC measurements could be used to predict risk for transplant-related events. Our main findings were 1) Higher TREC levels at 12 months were associated with superior overall survival, and 2) viral infections within the first 100 days after HSCT were associated with lower TREC levels.

TREC and KREC levels increased within the first three to six months after HSCT, suggesting T and B cell neogenesis in these intervals. This time to development of donor-derived lymphocytes was in line with previous studies (22, 24, 25) (**Figure 1**). The increase in TREC after three months might be aided by tapering of the T cell suppressive agents which is typically done at this time point for AML patients. It is known that the thymus involutes by age, and thus the ability to produce naïve T cells declines. Studies on HSCT patients as well as healthy populations showed an inverse correlation between TREC levels and age, more distinct in individuals under the age of 18 (35, 36). Although patient age was a strong determinant for TREC levels after HSCT in several previous studies, we only found a weak association (10, 20, 23, 24, 28, 37). Our cohort is more homogeneous and includes only adults, and the effect of age is therefore expectedly lower in the present study.

We demonstrated that patients who received ATG in addition to their conditioning regimen had lower TREC levels from HSCT until 12 months after the transplantation (**Table 2**). ATG was given to patients with a higher risk for GvHD and graft rejection, comprising all patients with a URD and only a few recipients of SIB (female donor to a male patient). ATG is a potent anti-T cell drug, and studies have shown that the drug can be detected in the bloodstream for several weeks to months after infusion (38, 39). The presented results are in line with earlier publications and add to the evidence that treatment with ATG can impair the neogenesis of T cells for several months post-HSCT (25, 37, 40). Factors thought to influence the specific patients’ immune reconstitution should be considered when determining the administration and dose of ATG. This said, ATG was not a significant variable for the development of infections (data not shown) or probability of survival (**Supplementary Table 1**) in our study, and thus a clear consequence of the delayed immune recovery was not evident for our cohort of adult AML patients.

Infections remain a serious threat to HSCT patients. Patients in our cohort who experienced viral infections or CMV reactivation had lower TREC levels 6 months after HSCT than those who did not (**Figure 2**). This is in line with earlier studies (21, 41, 42). However, the median time to first viral infection was about a month after HSCT. In the first months after HSCT, it is believed that most T cells in the recipient consist of peripherally expanded mature cells (10, 11). The significant rise in TREC levels was between three and six months in our cohort. Hence, most patients experienced the first viral infection before lymphocyte neogenesis had efficiently commenced. Studies have shown that the boost of CMV-specific CD8+ T cells due to CMV reactivation can delay the neogenesis of lymphocytes after HSCT (43). De König et al. likewise showed that Herpes virus 6 viremia was associated with impaired T cell immune reconstitution in children after HSCT due to the viral infection of CD4^+^ T cells (44). Other pathogens may similarly impact the level of T cells. Another possible effect of infections on TREC levels is the sole effect of dilution due to peripheral expansion of T cells. Hence, we observe an association between TREC levels and infections, but the causality needs further investigation.

Patients with higher TREC levels at the latest investigated time point had superior overall survival, in line with previous studies (21, 22, 25) (**Figure 3**). The main cause of death for our cohort was the relapse of leukemia. It is expected that a steady lymphocyte neogenesis would render a wider repertoire of T cell receptors and consequently cells with greater specificities against pathogens, but also against antigens of reoccurring malignant cells and hence stronger protection from relapse. It is known that the graft-versus-leukemia effect is very important for the long-term effect of HSCT. A potential explanation is that patients with delayed and impaired neogenesis of lymphocytes were less protected against relapse. Previous studies also demonstrated associations between lower TREC levels and relapse, as early as 2 months after HSCT (40, 45). Although not significant, we demonstrate a tendency toward superior RFS in patients with higher TREC levels 6 months after HSCT (p = 0.06, **Figure 3**). This finding emphasizes the importance of the detection of patients prone to relapse so that they can be kept under stringent control. Analysis of T cell reconstitution and neogenesis by TREC measurement could be considered as part of such detection in combination with other factors. Another study demonstrates that early CD4+ T cell engraftment can predict survival outcomes, which could be of valuable complement to the monitoring of TREC (46). Physicians may also consider introducing prophylactic measures such as tapering of immunosuppressants in selected patients. For patient post relapse we continued to study the levels of excision circles until the decision of palliative care, death, or re-transplantation. The acquired measurements were included in the survival analysis (**Figure 3**). This might be a potential bias while there is a possibility that these patients post relapse obtain treatment such as donor lymphocyte infusions with or without GvHD, chemotherapy, or discontinued immunosuppressive therapy. All are factors that could potentially influence TREC levels and survival at the landmark.

KREC levels were neither associated with the incidence of infections nor survival, in accordance with earlier studies (22, 25). Theoretically, T cells are expected to play a larger role in the defense against pathogens and recurrence of leukemia. T cell help is also warranted for full functionality of B cells, e.g., isotype switching of memory B cells and immunoglobulins. Our data hence confirm recent studies, that monitoring of KREC has less value than measurement of TREC in HSCT patients.

In summary, our results demonstrate that low TREC levels are associated with inferior survival and, earlier viral infections in adult AML patients after HSCT. We investigated the growing group of older HSCT recipients that could benefit from improved personalized evaluation of risk for various adverse outcomes. Our results suggest that specific evaluation of the immune reconstitution, namely T cell neogenesis by TREC measurement, could be considered as an additional part of the monitoring after HSCT for this subgroup of patients. This contribution to the risk-stratification might aid physicians to identify patients in need of closer follow-up, prolonged infection prophylaxis, and measures against relapse. Larger prospective studies are warranted to validate the findings of this study and elaborate on the optimal use of this marker in combination with conventional monitoring after HSCT.

## Data availability statement

The raw data supporting the conclusions of this article will be made available by the authors, without undue reservation.

## Ethics statement

The studies involving human participants were reviewed and approved by Swedish ethical review authority Stockholm, Sweden. Written informed consent for participation was not required for this study in accordance with the national legislation and the institutional requirements.

## Author contributions

MU, SV, and AS designed the study. KJ-V and SV performed the laboratory analyzes. SM and JT were responsible for the collection of clinical data. AS and HL performed data management and interpretation of data. AS was the main contributor to the data processing, analysis, and writing the manuscript. All authors contributed to the article and approved the submitted version.

## Funding

This work was supported by research funding from Knut and Alice Wallenbergs stiftelse, Cancerfonden, Barncancerfonden, Vetenskapsrådet, ALF Stockholm, and Radiumhemmets forskningsfonder.

## Acknowledgments

We would like to thank the personnel at the Department of Clinical Immunology and Transfusion Medicine, F79, Karolinska University Hospital, for help with the handling and analysis of samples. Also, thank you to Ahmed Gaballa and Tengyu Wang for your guidance in data management and statistical analysis.

## Conflict of interest

The authors declare that the research was conducted in the absence of any commercial or financial relationships that could be construed as a potential conflict of interest.

## Publisher’s note

All claims expressed in this article are solely those of the authors and do not necessarily represent those of their affiliated organizations, or those of the publisher, the editors and the reviewers. Any product that may be evaluated in this article, or claim that may be made by its manufacturer, is not guaranteed or endorsed by the publisher.
