# Supplementary material for: T cell receptor excision circles are potential predictors of survival in adult allogeneic hematopoietic stem cell transplantation recipients with acute myeloid leukemia
Source: Front Immunol. 2022 Sep 20;13:954716. doi: 10.3389/fimmu.2022.954716 (PMC9540498; doi:10.3389/fimmu.2022.954716)
Supplement: Supplementary file 1 [file DataSheet_1.docx]

Supplementary Material

# Supplementary Data

# Supplementary Figures and Tables


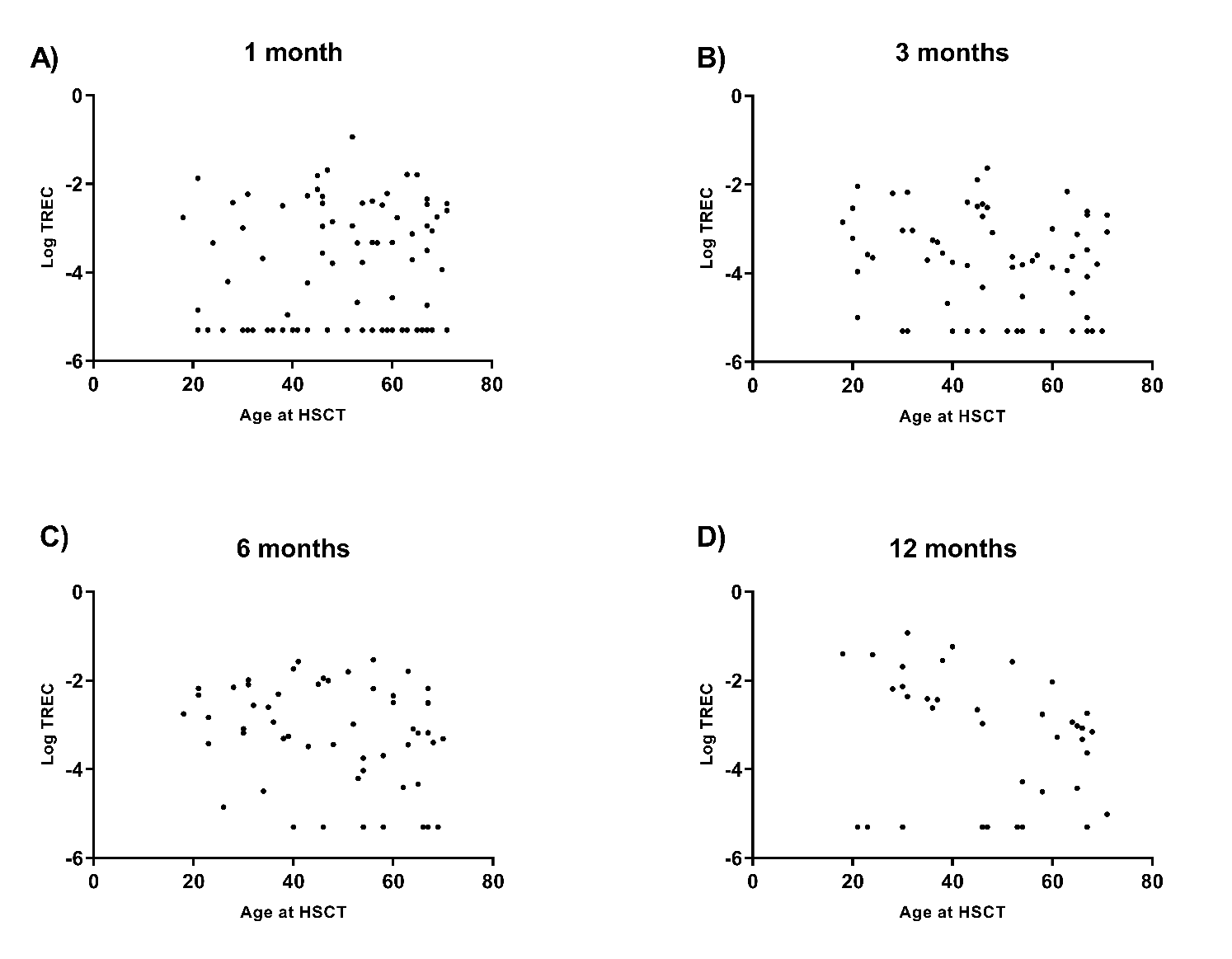


**Supplementary figure 1. No significant inverse correlation between patient age and TREC levels at any time points.** TREC levels at **A)** 1 month. R = 0.072, 95% CI: -0.15 – 0.29, p = 0.51 **B)** 3 months. R = -0.20, 95% CI: -0.43 – 0.058, p = 0.12 **C)** 6 months. R = -0.24, 95% CI: -0.48 – 0.04, p = 0.09 **D)** 12 months. R = -0.25, 95% CI: -0.52 – 0.075, p = 0.12. Spearman correlation.

| Outcome | Variables | HR | 95% CI | p-value |
| --- | --- | --- | --- | --- |
| Overall survival | Age patient | 1.00 | 0.98 - 1.03 | 0.81 |
|  | Age donor | 1.01 | 0.98 - 1.04 | 0.72 |
|  | cGvHD | 0.81 | 0.33 - 2.02 | 0.66 |
|  | ABO major mm | 1.26 | 0.54 - 2.92 | 0.59 |
|  | Severe aGvHD | 1.66 | 0.74 - 3.75 | 0.22 |
|  | ATG | 2.02 | 0.69 - 5.87 | 0.20 |
|  | CD34+ dose | 1.02 | 0.89 - 1.18 | 0.77 |
|  | Conditioning (MAC) | 0.89 | 0.39 - 2.01 | 0.77 |
|  | Sex (Male) | 0.99 | 0.45 - 2.19 | 0.99 |
|  | Sex F-M | 0.45 | 0.06 - 3.33 | 0.43 |
|  | TREC 12m | 0.52 | 0.34 - 0.81 | **0.004** |

Supplementary Table 1. TREC level at 12 months is the only significant variable associated with overall survival. Abbreviations; ABO major mm, major or bidirectional mismatch as opposed to ABO identity or minor mismatch; MAC, myeloablative conditioning as opposed to reduced-intensity conditioning; Sex F-M, female donor to male patient as opposed to all other sex matches. Cox proportional hazard regression, univariate analysis.
